# Modelling and Evaluating Electromagnetic Field Exposure in the Multiple-Source Scenario of Using IoT HF RFID Readers

**DOI:** 10.3390/ijerph19063274

**Published:** 2022-03-10

**Authors:** Patryk Zradziński

**Affiliations:** Laboratory of Electromagnetic Hazards, Central Institute for Labour Protection–National Research Institute (CIOP-PIB), Czerniakowska 16, 00-701 Warszawa, Poland; pazra@ciop.pl

**Keywords:** biomedical engineering, environmental engineering, induced (in situ) electric field strength, internet of things (IoT), numerical simulations, public health, specific energy absorption rate (SAR), RadioFrequency IDentification (RFID)

## Abstract

The aim of this study was to evaluate Specific Absorption Rate (SAR) and induced electric field (Ein) values in the model of a body of a person present near multiple HF RFID readers of a passive proximity integrated circuit card (PICC) working in an IoT application in a public transport vehicle, in order to test the hypothesis that even the simultaneous use of modelled readers does not cause electromagnetic field (EMF) exposure exceeding relevant limits provided for the evaluation of exposure of the general public. SAR and Ein values were evaluated under various exposure scenarios, designed to mimic EMF exposure under realistic conditions of HF RFID readers used on a public bus and covering various reader locations and the presence of a person using a PICC and a bystander. The results obtained from numerical modelling showed that the absorption of EMF emitted continuously by HF RFID readers (located 10 cm away from a body) in the human body may have a significant influence on humans when the PICC reading ranges are longer than 15–23 cm (depending on the class of PICC) for a single reader and when multiple sources of exposure are used in a public transport vehicle—even at reading ranges 15% shorter (13–20 cm).

## 1. Introduction

Constant technological development and the subsequent development of the digital society mean, among other things, that people’s daily lives and work environments involve more and more sources of radiofrequency electromagnetic field (RF-EMF) used for wireless communication between various devices. One of the most frequently used technologies for this purpose is High-Frequency (HF) RadioFrequency IDentification (RFID), working as a standalone system or as an integral part of a more sophisticated Internet of Things (IoT) system [1,2,3,4,5].

### 1.1. HF RFID Technology

HF RFID technology operates at frequency band 3–30 MHz, typically at 13.56 MHz. It is part of the globally used RFID technologies operating on various frequency bands, from 30 kHz up to 5.9 GHz [1,6]. HF RFID technology is used in the public environment and in many areas of the economy and medical centres [1,2,3,4,5]. Probably the most common application of HF RFID technology integrated in IoT systems today is managing proximity cards used in public transport, payment, access control to buildings or rooms, etc.

The operation of the HF RFID system is based on two groups of devices: readers/writers and passive tags. Passive tags, recognised by users as proximity cards or objects such as pendants, discs or bands (proximity integrated circuit card—PICC) includes an antenna that wirelessly collects the energy of EMF emitted by a nearby reader needed to energise a tag to be able to transfer back to a reader the data stored in the tag memory. The maximum distance at which a PICC can efficiently communicate with the reader is called the reading range (RR). In any particular HF RFID application, the RR is related to the strength of the magnetic field (H-Field, expressed in A/m) emitted from the reader and coming to the PICC location and the class of PICC used there. The ISO/IEC 14443-2:2020 standard distinguishes six classes of PICC devices requiring the influence of a magnetic field to be read of at least the following strengths (RH) [7]: devices of classes 1–3 require a minimum strength of magnetic field of RH(C1) = 1.5 A/m (1500 mA/m) to be sufficiently energised; class 4 ones require RH(C4) = 2.0 A/m (2000 mA/m); class 5—RH(C5) = 2.5 A/m (2500 mA/m) and class 6—RH(C6) = 4.5 A/m (4500 mA/m). The differences between the particular PICC classes are mainly related to the dimensions of the antennas built into them (the higher the class, the smaller the dimensions of the internal antenna, which in practice means its lower sensitivity to the influence of external EMF and the need to affect it with a stronger magnetic field to sufficiently energise the PICC).

The mentioned minimum levels of magnetic field required to sufficiently energise various PICC devices (RH) are stronger than the newest ICNIRP 2020 reference levels (RLicnirp2020): equal to 0.16 A/m (160 mA/m) regarding the protection of the general public against exposure to RF-EMF and 0.36 A/m (360 mA/m) regarding workers’ exposure (set to assess the whole-body EMF exposure as values averaged over 30 min) [8]. Additionally, in recent ICNIRP 2020 guidelines, reference levels to assess the local exposure (averaged over 6 min) were set to be equal to 0.36 A/m (360 mA/m) regarding the protection of the general public and 0.80 A/m (800 mA/m) regarding workers’ exposure—but still, they are lower than mentioned RH levels [8]. The older ICNIRP 1998 guidelines (still important because their limits were incorporated in various binding legislative documents, such as European Directive 2013/35/EU) that do not distinguish the time averaging approach between local and whole-body EMF exposure (both averaged over 6 min) set reference levels (RLicnirp1998) to be equal 0.073 A/m (73 mA/m) regarding the protection of the general public and 0.16 A/m (160 mA/m) regarding workers’ exposure [9]. The RH levels mentioned above are also higher than IEEE reference levels (RLieee2019): 1.2 A/m (1200 mA/m) regarding whole-body EMF exposure averaged over 30 min (lower than exposure required to energise tags at all PICC classes) and 2.68 A/m (2680 mA/m) regarding the local exposure averaged over 6 min (only PICC class 6 requires stronger exposure to energise its tags)—applicable both in unrestricted or restricted environments [10].

### 1.2. Example of HF RFID Technology Used in Public Environment

An example of a common multiple-source application of HF RFID technology working in the IoT system is the one used in public transport, e.g., buses, trams, city trains or the metro/subway.

**Figure 1:**
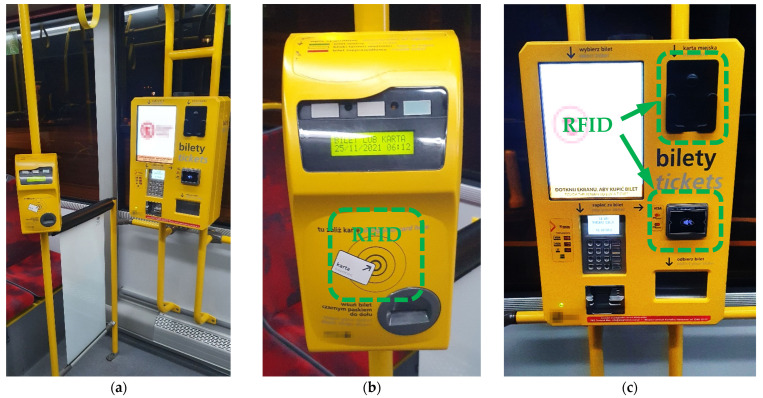
Examples of HF RFID readers used in public transport vehicles: (**a**) “ticket area” inside the central space of the bus; (**b**) ticket and contactless public transport card validator; (**c**) ticket machine operating the loading of contactless public transport cards using payment by a contactless card or other PICC electronic device (positions of HF RFID readers are marked with a dashed line).

The typical components of such a system are (1) ticket and contactless public transport card validators and (2) a ticket machine offering ticket coding on contactless public transport cards using payment with a contactless card or other PICC device integrated in an IoT system and managed by its main computer. Validators are located in various places in the vehicle, on vertical columns (usually in the passenger space) or attached to the vehicle cabin (with the centre of their HF RFID antenna located 1.0–1.2 m above the floor), while ticket machines are most often located in the middle of the vehicle passenger space, attached to the vehicle cabin (Figure 1). Using HF RFID readers in public transport typically does not require their direct contact with humans (except for contact with the hand holding the proximity card). However, due to the location of validators in the passenger space, especially during rush hours, passengers may find themselves very close to such devices, and in extreme cases may touch them with their body. In addition, a person coding a ticket on a public transport card or extending its expiry date (in a situation as shown in Figure 1a) may be exposed to EMF emitted simultaneously from three HF RFID readers (one operated in a validator and another two operated in a ticket machine). This is especially important because HF RFID readers built into validators emit EMF continuously, while in the case of ticket machines, the EMF emission is issued after inserting the PICC into the basket (photocell) or during the time dedicated to perform the necessary action with a proximity card or another PICC device used for a payment.

The internal antennas of an HF RFID reader used in such applications are typically rectangular in shape, with dimensions between 5 and 10 cm. The RR may be easily modified by changes in the reader settings in the output power of emitted EMF. Typically, the RR required for the proper functioning of the considered applications does not exceed a few centimetres, but tests performed near several ticket validators in the space of their regular use showed that real readers function with significantly longer RRs which even reached up to 15–18 cm.

Both contactless public transport cards and payment cards are usually PICC devices of classes 1–3, as defined following the relevant requirements of the ISO/IEC 14443-2:2020 standard [7].

### 1.3. Metrics Used to Evaluate the Direct Effects of Human Exposure to Radiofrequency EMF

A direct biophysical effect of the influence of RF-EMF with a frequency comparable to that emitted by HF RFID devices (13.56 MHz in most typical applications) is the absorption of electromagnetic energy in an organism and the thermal effects of this absorption. This interaction may be parameterised using what is known as the “specific energy absorption rate” (SAR), expressed in watts of absorbed EMF energy per kilogram of exposed tissue, W/kg [8,10]. International guidelines and standards provide SAR parameter definitions and limits regarding the evaluation of (1) general public exposure (ICNIRP)/exposure in unrestricted environments (IEEE), as well as 5-times-higher limits regarding the evaluation of (2) occupational exposure (ICNIRP)/exposure in restricted environments (IEEE) [8,10]. Three categories of SAR parameters and limits were provided by ICNIRP and IEEE:

- Whole-body averaged value (WBSAR), is averaged over 30 min—regarding general public exposure, the limit of this parameter is set at 0.08 W/kg (80 mW/kg);
- The local value in head and torso is averaged over 10 g cubic mass and also over 6 min—regarding general public exposure, the limit of this parameter is set at 2 W/kg (2000 mW/kg);
- The local value in limbs is averaged over 10 g cubic mass and also over 6 min—regarding general public exposure, the limit of this parameter is set at 4 W/kg (4000 mW/kg).

The results of the published research indicate a low variability of the ratio: SAR/EMF exposure level (up to 20% only) in the frequency band in which HF RFID readers operate (3–30 MHz) [11,12].

Regarding the HF RFID frequency band, international guidelines and standards (for EMF frequencies up to 10 MHz in the case of ICNIRP and up to 5 MHz in the case of IEEE) also require an assessment of the internal/in situ electric field (Ein) induced in an organism by EMF affecting humans—in order to prevent adverse effects to the nervous system. As mentioned above, HF RFID readers may operate in the frequency band 3–30 MHz, and the typical operating frequency of 13.56 MHz is similar to the above-mentioned upper frequencies for Ein evaluation. Due to the above-mentioned use of SAR and Ein limits in the HF RFID frequency band, Ein was also considered in this study. The published data presented above showed a low variation in SAR for frequencies 3–30 MHz at a fixed level of EMF exposure. Considering that SAR is proportional to the square of Ein, the variation in the Ein values covering this frequency range is expected to be even less than for SAR values. These Ein limits were provided separately regarding (1) general public exposure (ICNIRP)/exposure in unrestricted environments (IEEE) and higher limits regarding workers exposure: (2) limits 2 times higher than for the general public set by ICNIRP for occupational exposure and (3) equivalent limits 3 times higher than for unrestricted environments set by IEEE for exposure in restricted environments [8,10]. Ein values are not time averaged. According to ICNIRP, Ein values should be evaluated as root mean square (rms) values averaged over 2 mm × 2 mm × 2 mm contiguous tissue elements. Additionally, ICNIRP 2010 specifies that the 99th percentile value of the mentioned Ein spatial distribution over mass elements is the relevant parameter to be compared with the limits [13]. The frequency proportional limit of general public exposure (both ICNIRP 2020 and 2010) for its upper frequency of 10 MHz equals 1350 V/m, while extrapolated linearly from this frequency to a frequency of 13.56 MHz equals 1830 V/m (and 405–4050 V/m, covering the entire 3–30 MHz HF RFID frequency range). In turn, according to IEEE standards, the Ein values should be evaluated in the direction and location providing maximum Ein vector as rms values averaged over a 5 mm linear distance to be compared with the limits. The limit for exposure in unrestricted environments for its upper frequency of 5 MHz equals 1046 V/m, while extrapolated linearly from this frequency to a frequency of 13.56 MHz equals 2837 V/m (and 627–6277 V/m, covering the entire 3–30 MHz HF RFID frequency range). All of these Ein limits (ICNIRP and IEEE) were considered in this study.

### 1.4. The Aim

The aim of this study was to evaluate SAR and Ein values in the body of a person present in the “ticket area” of a public bus, near to a multiple-source HF RFID system—fixed HF RFID readers with passive PICCs (in proximity cards) working in the IoT system used in public transport—to test the hypothesis that even the simultaneous use of modelled readers does not cause EMF exposure exceeding relevant limits provided for the evaluation of exposure of the general public/a person in an unrestricted environment. Due to uniform electromagnetic principles of operating, we only looked at RFID readers using the HF band (3–30 MHz), regardless of whether they were a separate, standalone system or an integral part of the IoT system (such as: IoT wireless payment, automated fare collection or intelligent transport management systems).

SAR and Ein values were evaluated under various exposure scenarios, derived to mimic EMF exposure under realistic conditions of HF RFID readers used on a public bus.

## 2. Materials and Methods

### 2.1. Numerical Model of the EMF Source

**Figure 2:**
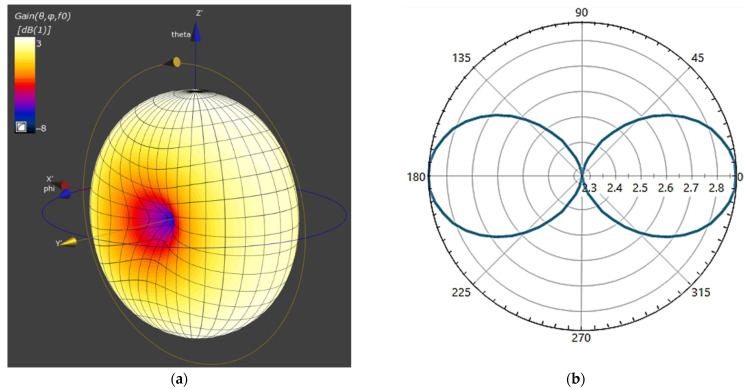
Investigated antenna far-field pattern: (**a**) 3D and (**b**) 2D in antenna plane.

The EMF source was a small (<λ/10) square-shaped loop antenna with inner dimensions of 80 × 80 mm and outer dimensions of 100 × 100 mm, modelled as a perfect electric conductor, radiating at 13.56 MHz. The dimensions of the modelled antenna are some of the largest dimensions used in HF RFID reader applications in public transport and were chosen to be the worst-case scenario of a source of EMF exposure, due to the higher EMF values at a particular distance from the reader, compared to readers using smaller antennas [2]. Initial input power to an antenna was set at 100 mW. The simulated omnidirectional antenna had a vertical radiation pattern and a far-field diagram in the shape of an eight (in the antenna plane) with a maximum total gain of 2.87 dB, as shown in Figure 2.

Elements related to the ticket validator with the HF RFID reader housing, its location and other electronic elements were not included in the numerical model.

**Figure 3:**
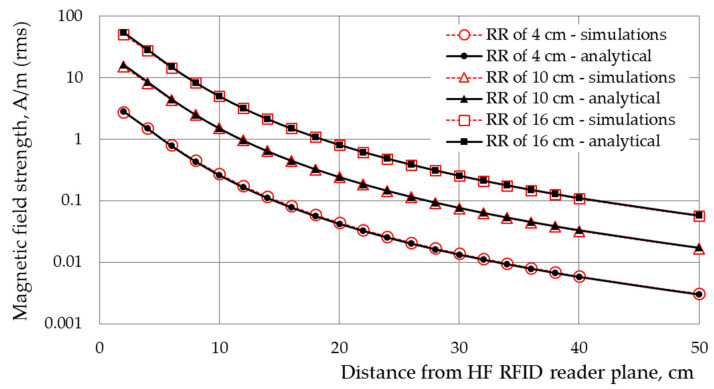
The EMF distribution (magnetic component), evaluated analytically and numerically (by Sim4Life software) and calculated along the axis perpendicular to the HF RFID reader with an antenna with inner dimensions of 8 × 8 cm, assuming that the output power of the reader is sufficient to ensure the RR of the considered reader: 4, 10 or 16 cm, according to ISO/IEC 14443-2:2020 requirements for PICCs of classes 1–3 (i.e., RH = 1.5 A/m).

The numerical model of the developed HF RFID antenna was validated by comparing the magnetic field strength distributions in front of the antenna, obtained by numerical simulations, with values calculated analytically for a rectangular conductor. The differences in magnetic field strength values numerically simulated and analytically calculated were found to be less than 10%, as shown in Figure 3.

### 2.2. Exposure Scenarios

**Figure 4:**
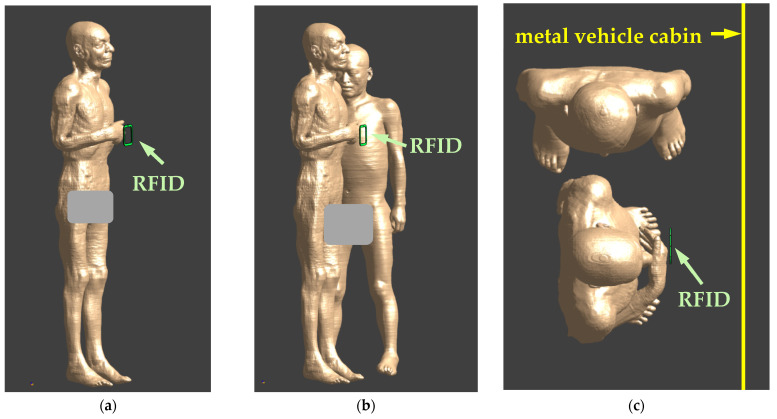
Exposure scenarios to EMF near HF RFID readers used in public transport vehicles: (**a**) side view of a person using the PICC device—reader on a vertical column; (**b**) side view of a person using the PICC device and a bystander (person standing nearby)—reader on a vertical column; (**c**) top view of a person using the PICC device and a bystander—reader attached to a vehicle cabin (the positions of the HF RFID readers are marked with arrows).

The investigations covered exposure scenarios representing realistic conditions for the interaction between a human body and the EMF emitted by a single HF RFID reader (equipped with a single antenna) used in a public bus (Figure 4).

Two typical locations of the HF RFID readers are considered:

- On a vertical column (typically in the passenger space)—modelled as an HF RFID located in a free space;
- Attached to the vehicle cabin—modelled as an HF RFID reader located next to the metal wall (made of 4 mm-thick steel).

In addition, in each of the above-mentioned exposure cases, the presence of one person (the person using the PICC device, i.e., validating the public transport card or coding a ticket, or extending the expiry date of the public transport card) or two people (the person using PICC device and a bystander) was considered.

In all the models of the investigated exposure scenarios, the centre of the HF RFID reader antenna was located at a height of 120 cm and at a distance of 10 cm to the closest surface of the torso of the model of the person validating the public transport card in front of it. This corresponds to the typical location of validators in public transport vehicles. In exposure scenarios with two people, the second person (bystander) was standing sideways to the person validating a public transport card at the shortest distance of the torso from the edge of the HF RFID reader of 25 cm, as shown in Figure 4.

### 2.3. Numerical Models of Human Body

The anatomical numerical male models Glenn and Jeduk, developed by the IT’IS (the Foundation for Research on Information Technologies in Society, Zurich, Switzerland), were used in the investigations. Both models are composed of over 300 tissues/organs and allow the body posture to be changed at the main joints of the body, e.g., knee, elbow, hip, shoulders or fingers. The Glenn model, with a height of 173 cm, a weight of 61.1 kg and a body mass index (BMI) of 20.4, was used as the person using the PICC device (proximity public transport card), while the Jeduk model with a height of 162 cm, a weight of 64.5 kg and a BMI of 24.6 was used as the bystander.

The dielectric parameters (at 13.56 MHz frequency) and densities of particular body tissues of which the Glenn and Jeduk numerical models are composed were extracted from the IT’IS Database for thermal and electromagnetic parameters of biological tissues [14].

### 2.4. Numerical Simulations

The numerical simulations were carried out using Sim4Life software (Zurich Med Tech, Zurich, Switzerland) using a Single EM FDTD (finite difference time-domain) solver. ABC (absorbing) boundary conditions were applied on all of the walls of the simulation domain except for the lower wall, where perfect electric conductor (PEC) boundary conditions were applied. The simulation domain (except for the lower wall and metal vehicle cabin) was extended by 1000 mm in each direction. The finest resolution used in the investigations was 1 mm set for the antenna and for the metal vehicle cabin and 2 mm set for the model of the human body. The resolution of the numerical models was finer than 1/15 of the wavelength in tissues at 13.56 MHz—the minimum resolution established for the evaluation of SAR required by the International Electrotechnical Commission (IEC) standard 62232:2017 [15]. The uncertainty of the numerical simulations (related to the models of field source and human body, type and location of the boundary conditions with respect to the model of the human body and position and the dielectric properties of the model of human body) was estimated as not exceeding ±25% (K = 1), within the range compliant with the state of the art in the field [15,16,17]. Local SAR10g values were calculated using the averaging algorithm according to IEC/IEEE 62704-1:2017 [16]. This algorithm is appropriate for use in a compliance assessment with the SAR limits established by IEEE and ICNIRP. To evaluate the worst-case EMF exposure scenario, the SAR simulations were performed for the continuous emission of EMF by HF RFID readers, with the PICC user remaining nearby over the entire averaging time (6 or 30 min) required by international standards and guidelines (ICNIRP and IEEE).

## 3. Results

**Figure 5:**
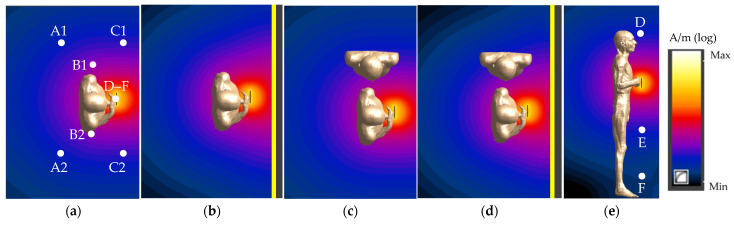
The normalised magnetic field distribution in a plane perpendicular to the centre of the HF RFID reader plane in various exposure scenarios: (**a**,**b**,**e**) the person using PICC device; (**c**,**d**) the person using PICC device and a bystander (reader on a vertical column (**a**,**c**,**e**) or attached to the vehicle cabin (**b**,**d**); horizontal (**a**–**d**) and vertical (**e**) cross-section; logarithmic scale).

**Figure 6:**
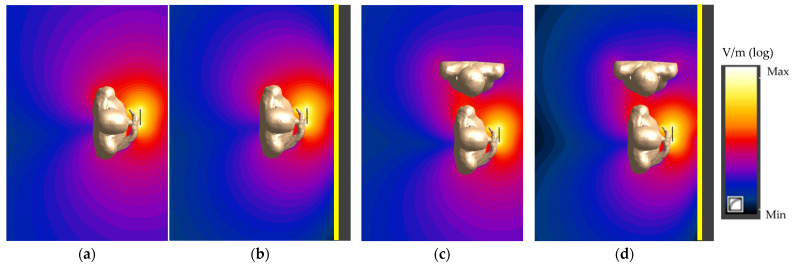
The normalised electric field distribution in a horizontal plane perpendicular to the centre of the HF RFID reader plane in various exposure scenarios: (**a**,**b**) the person using PICC device; (**c**,**d**) the person using PICC device and a bystander (reader on a vertical column (**a**,**c**) or attached to the vehicle cabin (**b**,**d**); logarithmic scale).

The presence of a metal vehicle cabin (modelled as a metal wall) and a person’s influence on the spatial distribution of EMF emitted by the HF RFID reader (especially in the surroundings of a bystander), as shown in Figure 5 and Figure 6 and Table 1 and Table 2, were analysed.

The electric and magnetic field values were evaluated in all the considered exposure scenarios and in the vicinity of RFID reader alone in the free space (i.e., in a numerical model with a reader on a vertical column, representing an unperturbed field distribution) or attached to the vehicle cabin, considering the output power of a reader sufficient to ensure the RR of 10 cm (according to ISO/IEC 14443-2:2020 requirements for PICCs of classes 1–3). The spatial distribution of the electric and magnetic fields was analysed in detail in nine points, as shown at Figure 5a,e:

- In a horizontal cross-section perpendicular to the reader plane (at a 120 cm height):
  – A1 and A2—behind the person using the PICC device to their left and right side, respectively (mirror image to the centre of the reader), in the bystander’s location;
  – B1 and B2—close to the left and right side of the person using the PICC device, respectively (mirror image to the centre of the reader), in the bystander’s location;
  – C1 and C2—in front of the person using the PICC device to their left and right side, respectively (mirror image to the centre of the reader), in the bystander’s location;
- Along a vertical line in the reader plane—D, E, F at heights of 170, 70 and 20 cm, respectively.

The highest variations of the point-values of the magnetic field in comparison to the unperturbed magnetic field values (from the model of the reader in a free space) were found in exposure scenarios with the reader attached to the vehicle cabin: an increase up to 40% (points C1 and C2) and a decrease up to 30% (points A1, B1, A2 and B2)—for points in the horizontal cross-section perpendicular to the centre of the reader plane, and an increase up to 50%—for points along the vertical line in the reader plane (Table 1). Corresponding to these, the increase in exposure scenarios with a reader on a vertical column were up to 15% and 20%, respectively. The increase (points A1 and B1) or decrease (point A2) related to the presence of a bystander in the modelled scenario did not exceed 10%.

A greater variation of the point values of the electric field than the magnetic field was found. The highest variations in the values of the electric field, in comparison to unperturbed electric field values (from the model of the reader in a free space), were found (contrary to the magnetic field) in exposure scenarios with the reader on a vertical column. Up to a 250% increase (points A1 and B1) and up to a 20% decrease (points C1, A2, B2 and C2)—for points in the horizontal cross-section perpendicular to the reader plane, and up to a 100% increase—for points along a vertical line in the reader plane—were found for them (Table 2). In exposure scenarios with the reader attached to the vehicle cabin, up to a 120% increase (points A1 and B1) and up to a 60% decrease (points C1, A2, B2, C2, D, E and F) were observed, respectively. The increase related to the presence of a bystander in the modelled scenario did not exceed 170%.

It should be noted that the increase in the reading range is related to a significant increase in the magnetic and electric field strengths at any point in the vicinity of the HF RFID reader. Comparing the investigated HF RFID reader (with inner dimensions of 80 × 80 mm and RR = 10 cm, corresponding to electric and magnetic field levels in its vicinity, as presented in Table 1 and Table 2), the values of the magnetic and electric field strength at particular locations were found to be lower for shorter RRs (e.g., 80% for an RR = 4 cm and 40% for an RR = 8 cm) and higher for longer RRs (e.g., 60%, 240% and 510% for RR of 12, 16 and 20 cm, respectively).

**Figure 7:**
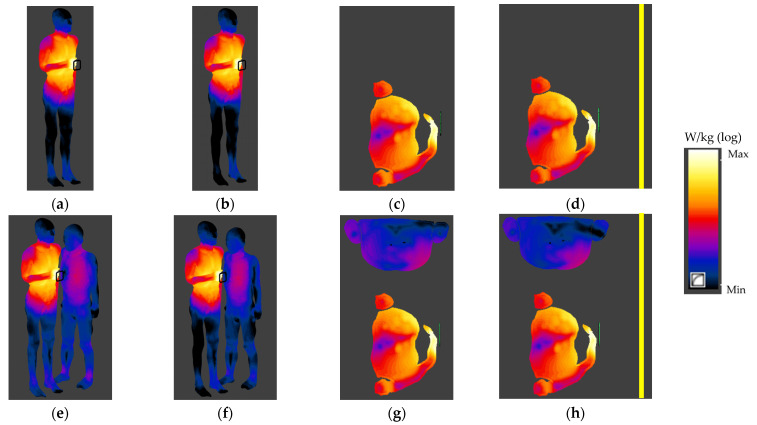
The normalised SAR distribution in the human body exposed to EMF near an HF RFID reader in various exposure scenarios: (**a**–**d**) a person using a PICC device; (**e**–**h**), a person using a PICC device and a bystander (side view (**a**,**b**,**e**,**f**) or horizontal cross-section perpendicular to the centre of the reader plane (**c**,**d**,**g**,**h**); reader on a vertical column (**a**,**c**,**e**,**g**) or attached to the vehicle cabin (**b**,**d**,**f**,**h**); logarithmic scale).

**Figure 8:**
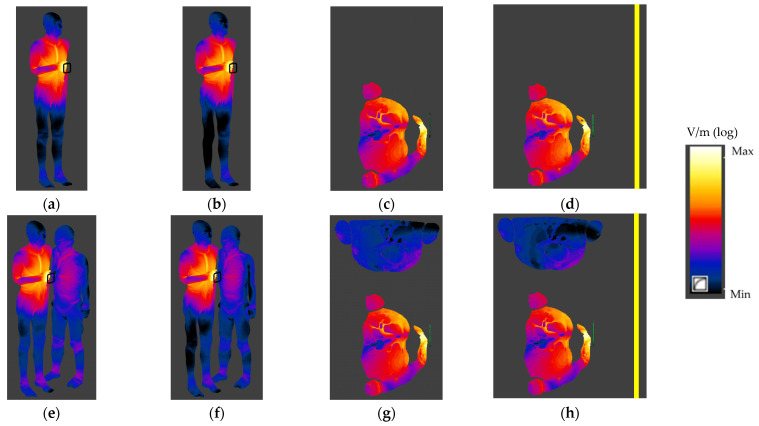
The normalised Ein distribution in the human body exposed to EMF near an HF RFID reader in various exposure scenarios: (**a**–**d**) a person using a PICC device; (**e**–**h**), a person using a PICC device and a bystander (side view (**a**,**b**,**e**,**f**) or horizontal cross-section perpendicular to the centre of the reader plane (**c**,**d**,**g**,**h**); reader on a vertical column (**a**,**c**,**e**,**g**) or attached to the vehicle cabin (**b**,**d**,**f**,**h**); logarithmic scale).

The normalised distributions of SAR and Ein values in the human bodies exposed to EMF near an HF RFID reader in various exposure scenarios (side view of values on the surface of the human body and values in the body horizontal cross-section perpendicular to the reader plane) are shown in Figure 7 and Figure 8.

Table 3 and Table 4 show the results of the numerical simulations of SAR and Ein values, respectively, related to exposure to EMF at 13.56 MHz near an HF RFID reader with an antenna with inner dimensions of 8 × 8 cm operating with an output power sufficient to ensure an RR of 10 cm (according to ISO/IEC 14443-2:2020 requirements for PICCs of classes 1–3). Results were obtained with respect to continuous exposure (typical for validators) and worse-case exposure, associated with respect to the exposure duration considered in the SAR averaging time required by international guidelines and standards (6 min for local SAR10g and 30 min for WBSAR). It is also worse-case exposure when Ein is considered without time-averaging.

The highest SAR values were found in the model of a person using a PICC device in an exposure scenario with two people and an HF RFID reader (validator) located on a vertical column—7.4 mW/kg for SAR10g in head and torso, 46 mW/kg for SAR10g in limbs and 0.28 mW/kg for WBSAR (Table 3). No significant differences between SAR values in the model of a person using a PICC device obtained for all the investigated exposure scenarios were found—all observed differences were below the level of uncertainty for the carried out numerical simulations, estimated as ±25% (K = 1). The highest differences of 5% were found between values obtained for exposure scenarios with various HF RFID reader locations (on a vertical column and attached to the metal vehicle cabin). In the case of a bystander, the highest SAR values were found in the same exposure scenario where the highest SAR values were found for the person validating a public transport card—0.07 mW/kg for SAR10g in the head and torso, 0.06 mW/kg for SAR10g in limbs and 0.002 mW/kg for WBSAR. These values were at least 100 times lower than the SAR values mentioned above, obtained in the model of a person using a PICC device, and up to 2 times higher than SAR values obtained for an HF RFID reader attached to the metal vehicle cabin.

All other SAR values in both human body models (of a person using a PICC device and the bystander) were below 1.2% of the limits of the general public (GP) exposure (ICNIRP 2020)/exposure in unrestricted environments (IEEE), and thus the limits of occupational exposure (OE) (ICNIRP)/exposure in restricted environments (IEEE).

The highest peak Ein values were found in the model of a person using a PICC device and were found also in the same exposure scenario as for the highest SAR values—55 V/m and 49 V/m, calculated according to ICNIRP and IEEE requirements, respectively, as shown in Table 4. Additionally, in the case of the Ein analysis, no significant differences (up to 15% for various HF RFID reader locations) between the values in the model of a person using a PICC device obtained for all investigated exposure scenarios were found—all differences were below the level of uncertainty of the numerical simulations carried out, estimated as ±25% (K = 1). The 99th percentile value of Ein in all human body model tissues were up to 11 times lower than peak Ein values. Additionally, the Ein values calculated according to ICNIRP requirements were up to 20% higher than the values calculated according to IEEE requirements.

In the case of the bystander, the highest Ein values were found in the same exposure scenario as for a person using a PICC device—2.0 V/m and 1.5 V/m, calculated according to ICNIRP and IEEE requirements, respectively, as shown in Table 4. These values were up to 45 times lower than the Ein values mentioned above, obtained in a model of a person using a PICC device, and up to 40% higher than the Ein values obtained for an HF RFID reader attached to the metal vehicle cabin. The 99th percentile values of Ein in all human body model tissues were up to 7 times lower than peak Ein values. Additionally, the Ein values calculated according to ICNIRP requirements were up to 40% higher than the values calculated according to IEEE requirements.

The highest Ein values (shown in Table 4) in the model of a person using a PICC device reached up to 5% of the limits of GP and up to 2% of the limits off OE. All Ein values in the model of the bystander were below 1% of these limits.

## 4. Discussion

Various HF RFID applications in IoT systems are used with different RRs (typically from a few up to several centimetres, depending on particular application needs and the individual settings of output power in readers), as well as the use of PICCs of various classes (from 1 to 6, according to ISO/IEC 14443-2:2020).

Table 5 and Table 6 present the results of the numerical simulations of SAR and Ein values, respectively, related to the exposure of a person using a PICC device to EMF at 13.56 MHz near to the HF RFID reader with an internal antenna with inner dimensions of 8 × 8 cm, assuming that the RR of the considered reader is 4, 10 or 16 cm and used various PICCs devices (of classes 1–6). The maximum RRs for which SAR or Ein values are compliant with limits of general public exposure (ICNIRP) or limits of exposure in unrestricted environments (IEEE) are also shown.

The SAR values calculated for systems with RRs of 10 and 16 cm were 30 times and 330 times higher than the SAR values calculated for an RR of 4 cm (Table 5). Additionally, SAR values up to 2, 3 and 9 times higher were found for PICCs of classes 4, 5 and 6, respectively, compared to the values corresponding to the use of PICCs of classes 1–3.

It was found that only the model of a person using a PICC device of classes 5 and 6, in the system with 16 cm RR, experienced WBSAR and local SAR10g in head and torso values reaching up to 12% and 37%, respectively, of the limits of GP (shown in Table 5). For those cases, the WBSAR and local SAR10g in head and torso values did not exceed 10% of the limits of OE. Additionally, local and SAR10g in limbs exceeding 115% of the limits of GP was only found in a person using a PICC device of class 6 in the system with 16 cm RR (23% of the limits of OE). For other cases in the system with 16 cm RR, those values reached 13%; 23% and 35% of the limits of GP for PICC devices of classes 1–3; 4 and 5, respectively. The values obtained for other investigated cases did not exceed 10% of the limits of GP and OE.

The maximum RRs for which SAR values were compliant with limits of GP exposure were estimated to be between approximately 23 cm (when the most common PICCs of classes 1–3 are used) and 15 cm (when PICCs of class 6 are used). The results of this study showed that SAR values in the body exposed to EMF at 10 cm away from HF RFID readers (in exposure scenarios similar to those considered in this study) may exceed the limits of GP exposure, when the RR exceeds 15 cm (taking into account continuous exposure over a time when anyone stays for longer than 6 min near the validator of continuous RF-EMF emission). Such a case of exposure is especially possible during rush hour and does not have to be related to the exposure while validating a public transport card. In cases of an exposure duration shorter than 6 min, the SAR values will be proportionally lower. However, it should be noted that, in an extreme case, the human body may even touch an HF RFID reader (a case of exposure not covered by this study) for which SAR values (especially local ones) may be significantly higher than those presented in this study. Additional numerical simulations are required to take that extreme case of exposure into consideration.

Moreover, the magnetic field strengths in the location of a person using a PICC device (undisturbed by the presence of a human) do exceed the reference levels limits of GP exposure in most of the considered exposure scenarios (and in many cases, the limits of OE are exceeded), but the SAR values are compliant with the limits, except for the exposure case when PICCs of class 6 are used in the system with an RR exceeding 15 cm.

The Ein values calculated for systems with RRs of 10 and 16 cm were up to 6 times and 18 times higher than Ein values calculated for an RR of 4 cm (Table 6). Additionally, up to 30%, 70% and 200% higher Ein values were found for the use of PICCs of classes 4, 5 and 6, respectively, compared to the values corresponding to the use of PICCs of classes 1–3.

The Ein values (shown in Table 6) in the model of a person using a PICC device of classes 1–6 in systems with 4 and 10 cm RRs did not exceed 10% of the limits of GP and OE, except for the PICC device of class 6 and 10 cm RR, for which they reached up to 15% of the limits of GP. In the case of a system with a 16 cm RR, Ein values reached up to 15%, 20%, 25% and 45% of the limits of GP and up to 7%, 9%, 12% and 20% of the limits of OE for the use of a PICC device of classes 1–3, 4, 5 and 6, respectively.

The maximum RRs for which Ein values are compliant with the limits of general public exposure (ICNIRP) or limits of exposure in unrestricted environments (IEEE) was estimated:

- Between approximately 32 cm (most common PICCs of classes 1–3) and 22 cm (PICCs of class 6)—when considering ICNIRP 2020, peak values and limits for 10 MHz;
- Between approximately 36 cm (PICCs of classes 1–3) and 25 cm (PICCs of class 6)—when considering ICNIRP 2020, peak values and limits extrapolated linearly for 13.56 MHz;
- Between approximately 72 cm (PICCs of classes 1–3) and 50 cm (PICCs of class 6)—when considering ICNIRP 2010, 99th percentile values and limits for 10 MHz;
- Between approximately 80 cm (PICCs of classes 1–3) and 55 cm (PICCs of class 6)—when considering ICNIRP 2010, 99th percentile values and limits extrapolated linearly for 13.56 MHz;
- Between approximately 46 cm (most common PICCs of classes 1–3) and 31 cm (PICCs of class 6)—when considering IEEE, peak values and limits for 5 MHz;
- Between approximately 63 cm (most common PICCs of classes 1–3) and 42 cm (PICCs of class 6)—when considering IEEE, peak values and limits extrapolated linearly for 13.56 MHz.

The results of this study showed that Ein values in a body exposed to EMF at a distance of 10 cm away from HF RFID readers in exposure scenarios similar to those considered in this study do not exceed the limits of GP exposure for typical reading ranges (up to 15–18 cm). Additionally, in the case of Ein assessment, extreme cases of exposure when the human body touches the HF RFID reader requires further investigation.

In public transport, any person present in the “ticket area” may be exposed to RF-EMF from three HF RFID readers, as shown at Figure 1a. Such a case (called multiple-source exposure), along with cases of multifrequency exposure according to international guidelines and standards (ICNIRP, IEEE and IEC 62232:2017), needs to be included in the SAR and Ein assessment [8,10,13,15]. Guidelines on how to assess this exposure, depending on whether it is correlated or uncorrelated in time, are provided by IEC Technical Report 62630:2010 or ISO/IEC 14443-2:2020 [15,18], for example. According to these guidelines, under exposure from multiple sources uncorrelated in time, such as in the “ticket area”, SAR is an algebraic sum at any point in the body (mass element) of SAR values calculated separately for each considered source. At the worst theoretic case, assuming that all RF-EMF sources are in adjacent, very close locations, SAR and Ein values will be many times higher, as there are sources taken into account in the assessment, i.e., in the case of a person present in the “ticket area”—3 times higher (three HF RFID readers) than the values presented in this study. The maximum RRs for which SAR and Ein are compliant with the limits of GP, exposure will be approximately 15% and 30% shorter, respectively, than corresponding maximum RRs for a single HF RFID reader in this study (as shown in Table 5 and Table 6). However, under real (typical) exposure conditions of a person present in the “ticket area”, the SAR and Ein values in real environmental situations are expected to be similar to those presented above for a single reader of continuous EMF emissions (i.e., the location of readers by various parts of the body, greater distances among readers than their dimensions, continuous EMF emissions from readers built into the validator and short EMF emissions (from several seconds to tens of seconds) from readers built into ticket machines).

ISO/IEC 14443-2:2020 [7] distinguishes two communication signal interfaces: types A and B. According to this standard, communications from HF RFID readers to PICC devices requires Amplitude Shift Keying (ASK) in 100% or 10% modulations. In the case of communications from PICC devices to HF RFID readers, other modulations are used, e.g., Binary Phase Shift Keying (BPSK) or On/Off Keying (OOK) as well as various coding such as Non-Return to Zero (MRZ) or Manchester, respectively. Furthermore, in communications from the reader to the PICC device, an ASK modulation of 2–3 μs duration should be used. The ratio between modulated and unmodulated periods of transmission time depends on the ratio of “1” and “0” in data to be transmitted. The duration of the transmission depends on data size and transmission rate. Another important parameter is the duty cycle. This depends on the purpose of the HF RFID system application and ranges from 0.1 to 0.8 according to the Electronic Communications Committee (ECC) report 208 [19]. All these parameters influence the value of the emitted power averaged over time and thus SAR values (time averaged). The parameters influencing the SAR values the most are ASK 100% modulation and the duty cycles mentioned above. The SAR values should be 10 times, 2 times and 20% lower than the values presented in this study for continuous RF-EMF emissions when duty cycles of 0.1, 0.5 and 0.8 are considered, respectively. The maximum RRs for which SAR is compliant with the limits of GP exposure will be approximately 50%, 15% and 5% longer for duty cycles of 0.1, 0.5 and 0.8, respectively, than corresponding to maximum RRs for the HF RFID reader in this study.

However, it must be pointed out that the maximum RRs for which the considered Ein is compliant with the limits of GP exposure remain at the same level, because Ein values are not time averaged. Additionally, it was found that for low-duty cycles (e.g., 0.1), the maximum RRs for which Ein is compliant with the limits of GP exposure are shorter than RRs for which SAR is compliant with these limits. This justifies the need to evaluate not only SAR limits compliance but also assess the Ein compliance with limits under RF-EMF exposure from HF RFID readers. A detailed analysis of the impact of the communication parameters, the modulations, duty cycles, etc., also requires further investigation.

If HF RFID readers equipped with antennas with dimensions smaller than the dimensions used in this study are used in IoT systems, both the SAR and Ein values (both in the person validating the public transport card 10 cm away from HF RFID reader and the bystander) will be lower than those presented in this article, though anyone in direct proximity to the emitting antennas may be exposed to higher values.

To take another point of view, it is worth mentioning the work currently underway related to the application of IoT systems using HF RFID technology that does not require the PICCs to be brought near the readers (payment for journeys will be charged, for example, on the basis of identifying a proximity payment card, e.g., in a pocket of a passenger passing nearby the reader). Such a solution would require a significant increase in RRs, and thus an increase in exposure to the EMF emitted by them, along with an increase in the SAR and Ein values in people near to the readers.

It should be noted that people using public transport may also be users of active implantable medical devices (AIMDs) such as hearing implants, cardiac pacemakers, implantable cardioverter-defibrillators, insulin pumps, etc. Following requirements regarding the protection of the public and workers against electromagnetic hazards, potential malfunctions of AIMDs exposed to EMF from HF RFID readers need to be taken into consideration [20,21]. For example, published studies show higher SAR values in tissues next to AIMDs (their metal elements) compared to the SAR values calculated in the same tissues of a person who is not a user of such implants [2,22,23,24,25]. Such an effect decreases the maximum RRs that may be counted to ensure that the general public limits of SAR and Ein are not exceeded in anyone present near HF RFID readers. Taken together, despite the results of my studies, which suggest that today, the EMF exposure in the ticket area of public busses seems to be typically compliant with relevant limits of general public exposure, further development in the applications of this technology also needs attention to the evaluation of the effects of EMF exposure, as well as attention to the possible technical measures reducing the EMF influence on anyone present nearby readers (especially when the use of readers of higher EMF emission is considered, even when today, the frequency of 13 MHz is not covered by the regular EMC requirements applicable for AIMD).

## 5. Conclusions

The main contribution of this work is that it shows that absorption in the human body of RF-EMF emitted continuously by a single HF RFID reader working in the IoT system in public transport vehicles may have a significant influence on humans when it is used in the system with RRs longer than approximately 15 cm, with PICCs devices of class 6 or longer than approximately 23 cm, with the most common PICCs devices of classes 1–3. It has also been shown that, in the bus ticket area, where having multiple sources of exposure is common (from three RFID readers installed there), the RF-EMF absorption in the human body may have a significant influence on anyone present there when RRs are 15% shorter than in the case of a single reader.

It should also be pointed out that, under RF-EMF exposure from HF RFID readers operating at any frequency from the HF band (3–30 MHz frequency), in exposure scenarios similar to those considered in this study (the body of person using a PICC device exposed to RF-EMF with the torso and hand holding the PICC device 10 cm and 0.5 cm away from the HF RFID, respectively, and a bystander present near to the HF-RFID-emitting antennas), the compliance assessment with public exposure limits of both SAR and Ein should be considered for low-duty cycles of RF-EMF emissions (at a level of 0.1).

This study indicates that the presence of metal objects, such as vehicle cabin, as well as people present in the vicinity of HF RFID readers can have an influence on the distribution of EMF emitted by the reader.

In this work, the main contribution concerned the HF RFID readers used in public transport vehicles, but similar devices are widely used in many other businesses, such as offices, shops, factories, medical centres and so on, and RF-EMF exposure and electromagnetic hazards in their vicinity are expected to be similar to that presented in this study.

## Figures and Tables

**Table 1:** Magnetic field strength values in various exposure scenarios near to the HF RFID reader with an internal antenna of inner dimensions of 8 × 8 cm, operating with an output power sufficient for an RR of 10 cm (according to ISO/IEC 14443-2:2020 requirements for PICCs of classes 1–3).

Exposure Scenario	Magnetic Field Strength, mA/m								
	Evaluation Points								
	A1	B1	C1	A2	B2	C2	D	E	F
Reader alone on vertical column	7.0	41	12	7.0	41	12	8.0	8.0	1.1
One person and reader on vertical column	7.0	39	12	8.0	42	12	8.2	9.1	1.3
Two persons and reader on vertical column	7.3	42	12	7.1	42	12	8.2	9.0	1.3
Reader alone attached to vehicle cabin	5.1	38	17	5.1	38	17	10	10	1.0
One person and reader attached to vehicle cabin	5.3	35	17	5.5	38	17	11	12	1.0
Two persons and reader attached to vehicle cabin	5.3	37	17	5.3	38	17	11	11	1.1

**Table 2:** Electric field strength values in various exposure scenarios near to the HF RFID reader with an internal antenna of inner dimensions of 8 × 8 cm, operating with an output power sufficient for an RR of 10 cm (according to ISO/IEC 14443-2:2020 requirements for PICCs of classes 1–3).

Exposure Scenario	Electric Field Strength, V/m								
	Evaluation Points								
	A1	B1	C1	A2	B2	C2	D	E	F
Reader on vertical column	0.23	1.0	0.60	0.23	1.0	0.60	0.55	0.38	0.11
One person and reader on vertical column	0.30	1.3	0.60	0.21	0.92	0.57	0.66	0.39	0.16
Two persons and reader on vertical column	0.80	2.3	0.49	0.22	0.93	0.53	0.70	0.47	0.22
Reader alone attached to vehicle cabin	0.17	0.92	0.37	0.17	0.92	0.37	0.28	0.19	0.046
One person and reader attached to vehicle cabin	0.22	1.1	0.31	0.16	0.79	0.29	0.37	0.27	0.079
Two persons and reader attached to vehicle cabin	0.51	1.8	0.57	0.15	0.79	0.28	0.50	0.30	0.11

**Table 3:** SAR values in the human body models in various exposure scenarios related to exposure to EMF at 13.56 MHz near to the HF RFID reader with an internal antenna with inner dimensions of 8 × 8 cm, assuming that the RR of considered reader is 10 cm (according to ISO/IEC 14443-2:2020 requirements for PICCs of classes 1–3).

Exposure Scenario	Person under Exposure Evaluation	WBSAR ^1^, mW/kg	SAR10g ^2^, mW/kg	
			Head/Torso	Limb
One person and reader on vertical column	PICC device user	0.28	7.4	46
Two persons and reader on vertical column	PICC device user	0.28	7.4	46
	Bystander	0.0024	0.070	0.060
One person and reader attached to vehicle cabin	PICC device user	0.27	7.1	46
Two persons and reader attached to vehicle cabin	PICC device user	0.28	7.1	46
	Bystander	0.0016	0.038	0.031

^1^ WBSAR–SAR evaluated as averaged over the entire exposed body; ^2^ SAR10g—maximum local SAR averaged over a 10 g cubic massof tissue.

**Table 4:** Ein (internal/in situ electric field) values in the human body models in various exposure scenarios related to exposure to EMF at 13.56 MHz near to the HF RFID reader with an internal antenna with inner dimensions of 8 × 8 cm, assuming that the RR of considered reader is 10 cm (according to ISO/IEC 14443-2:2020 requirements for PICCs of classes 1–3).

Exposure Scenario	Person under Exposure Evaluation	Ein ICNIRP ^1^, V/m		Ein IEEE ^2^, V/m	
		Peak	99th Perc	Peak	99th Perc
One person and reader on vertical column	PICC device user	52	5.0	43	4.7
Two persons and reader on vertical column	PICC device user	55	5.1	49	4.8
	Bystander	2.0	0.30	1.5	0.26
One person and reader attached to vehicle cabin	PICC device user	52	5.0	43	4.3
Two persons and reader attached to vehicle cabin	PICC device user	55	5.0	49	4.7
	Bystander	1.5	0.25	1.1	0.24

^1^ Ein ICNIRP—internal electric field values averaged as rms values over 2 mm × 2 mm × 2 mm contiguous tissue, 99th perc—99th percentile value of Ein; ^2^ Ein IEEE—in situ electric field value evaluated in the direction and location providing maximum Ein vector as rms values averaged over a 5 mm linear distance in tissues.

**Table 5:** SAR values in the model of a person validating a public transport card in an exposure scenario with two people near an HF RFID reader (at 13.56 MHz) with an internal antenna with inner dimensions of 8 × 8 cm, assuming that the RR of considered reader is 4, 10 or 16 cm (according to ISO/IEC 14443-2:2020 requirements for PICCs of various classes).

PICC Class ^1^	RR, cm	WBSAR ^2^, mW/kg	SAR10g ^3^, mW/kg		Maximum RR When Exposure is Compliant with GP Limits ^4^, cm
			Head/Torso	Limb	
1–3	4	0.0094	0.25	1.5	23
	10	0.28	7.4	46	
	16	3.2	83	510	
4	4	0.017	0.44	2.7	21
	10	0.50	13	81	
	16	5.6	150	900	
5	4	0.026	0.69	4.2	19
	10	0.79	21	130	
	16	8.7	230	1400	
6	4	0.085	2.2	140	15
	10	2.5	6.7	410	
	16	28	740	4600	

^1^ PICC class—proximity integrated circuit card (PICC) class according to ISO/IEC 14443-2:2020 differing in minimum magnetic field required to energise and read PICCs and the dimensions of PICCs; ^2^ WBSAR—SAR evaluated as averaged over the entire exposed body; ^3^ SAR10g—the maximum local SAR averaged over a 10 g cubic mass of tissue; ^4^ the maximum RR when exposure is compliant with the GP limits—the maximum RR when the level of emitted EMF does not cause SAR values exceeding the limits of general public exposure (ICNIRP) or limits of exposure in unrestricted environments (IEEE).

**Table 6:** Ein values in the model of a person validating a public transport card in an exposure scenario with two people near to the HF RFID reader (at 13.56 MHz) with an internal antenna with inner dimensions of 8 × 8 cm, assuming that the RR of the considered reader is 4, 10 or 16 cm (according to ISO/IEC 14443-2:2020 requirements for PICCs of various classes).

PICC Class ^1^	RR, cm	Ein ICNIRP ^2^, V/m		Ein IEEE ^3^, V/m		Maximum RR When Exposure is Compliant with GP Limits ^4^, cm			
		Peak	99th Perc	Peak	99th Perc	ICNIRP Peak/99th Perc of Ein		IEEE Peak	
						@10 MHz	@13.56 MHz	@5 MHz	@13.56 MHz
1–3	4	10	0.93	8.9	0.87	32/72	36/80	46	63
	10	55	5.1	49	4.8				
	16	180	17	160	16				
4	4	13	1.2	12	1.2	29/66	32/75	40	58
	10	73	6.8	65	6.3				
	16	250	23	220	21				
5	4	17	1.5	15	1.5	27/61	30/65	38	53
	10	92	8.4	81	7.9				
	16	310	28	270	26				
6	4	30	2.8	27	2.6	22/50	25/55	31	42
	10	170	15	150	14				
	16	550	51	490	48				

^1^ PICC class—proximity integrated circuit card (PICC) classes according to ISO/IEC 14443-2:2020 differing in minimum magnetic field required to energise and read PICCs and dimensions of PICCs; ^2^ Ein ICNIRP—internal electric field values averaged as rms values over 2 mm × 2 mm × 2 mm contiguous tissue, peak—peak spatial value of Ein required by ICNIRP 2020, 99th perc—99th percentile value of Ein required by ICNIRP 2010; ^3^ Ein IEEE—in situ electric field value evaluated in the direction and location providing the maximum Ein vector as rms values averaged over a 5 mm linear distance in tissues; ^4^ the maximum RR when exposure is compliant with the GP limit—the maximum RR when the level of emitted EMF does not cause Ein values exceeding the limits of general public exposure (ICNIRP) at 10 MHz or extrapolated linearly for 13.56 MHz or exposure in unrestricted environments (IEEE) at 5 MHz or extrapolated linearly for 13.56 MHz.
